# An integrative approach between neurodiversity perspectives and quality of life models for autistic people across the spectrum of support needs

**DOI:** 10.3389/fpsyt.2025.1756323

**Published:** 2026-01-05

**Authors:** Jon Liñares-de-Marcos, Blanca Palomero-Sierra, Victoria Sánchez-Gómez, Clara J. Fernández-Álvarez, Ricardo Canal-Bedia

**Affiliations:** 1 Institute for Community Inclusion (INICO), University of Salamanca, Salamanca, Spain; 2 Institute for Biomedical Research of Salamanca (IBSAL), University of Salamanca, Salamanca, Spain; 3 Department of Basic Psychology, Psychobiology, and Behavioral Science Methodology, University of Salamanca, Salamanca, Spain; 4 Department of Personality, Assessment, and Psychological Treatments, University of Salamanca, Salamanca, Spain

**Keywords:** autism, identity, inclusive research, neuroaffirmative approach, neurodiversity, quality of life, self-advocacy, supports

## Abstract

**Introduction:**

Autism is increasingly understood not through deficit-based frameworks but through approaches that emphasize rights, inclusion, and well-being for autistic people across the spectrum of support needs, including non-speaking individuals, those with intellectual disability, and those experiencing mental health challenges. Two perspectives have been central to this shift: Quality of Life (QoL) models, rooted in applied disability research, and the neurodiversity paradigm, arising from autistic self-advocacy and social justice movements.

**Aim:**

This mini review examines the convergences and tensions between these perspectives, generating a set of integrative principles to guide support providers, researchers, and policymakers. Evidence is synthesized across three thematic perspectives: socio-political and paradigmatic debates, particularly language, identity, and representation; applied and clinical practice, including the aims, role, and risks of supports and interventions; and research, with attention to participatory approaches, lived-experience priorities, and the representation of autistic people with extensive support needs.

**Discussion:**

Six principles emerge: (i) well-being depends on both self-acceptance and the quality of supports; (ii) language should balance contextual function with individual preference; (iii) identity has transformative value, requiring diagnostic practices that are inclusive, participatory, and non-deficit oriented; (iv) supports are essential mechanisms for participation, not threats to identity; (v) interventions should promote autonomy, belonging, and growth without enforcing normalization; and (vi) research must ensure autistic participation across all stages, with accessible processes and priorities aligned with autistic preferences. Together, these principles offer a framework for integrating QoL and neurodiversity approaches in ways that advance rights, inclusion, and well-being.

## 1 Introduction

Autism is understood as a heterogeneous condition, encompassing a wide range of cognitive and communication profiles, sensory orientations, and everyday adaptive behavior characteristics. This highlights the substantial variability observed across autistic populations and underscores the importance of approaches that aim to understand what enables well-being and inclusion within this diversity (1). Over the past three decades, this focus has gradually shaped disability policy and practice, consistent with the principles of the United Nations Convention on the Rights of Persons with Disabilities (UNCRPD) (2), which emphasize the realization of rights as a fundamental guiding principle.

Within this context, comprehensive models that integrate rights and the provision of supports to enhance quality of life (QoL) have provided operational tools to pursue these aims. QoL refers to an individual’s evaluation of their goals and expectations in relation to their life circumstances, considering the cultural context and value systems involved (3). It is a multidimensional construct with both objective indicators and subjective perceptions, and is achieved when personal needs are met and opportunities for participation in various life contexts are available (4). Within the disability field, QoL-centered models adopt a socioecological and interactionist perspective, conceptualizing disability as arising from the dynamic interplay between personal characteristics, environmental demands, and available supports (5).

In parallel, the neurodiversity paradigm emerged from neurodivergent self-advocacy and activism (6). It reframed autism as a natural form of human variation and expanded the notion of well-being to include belonging, authenticity, and self-acceptance (7). This perspective rejects pathologizing interpretations and foregrounds identity, rights, and self-representation, promoting neurodivergent pride and empowerment through social justice (8). Beyond its conceptual proposal, the neurodiversity paradigm constitutes a form of social and identity politics that challenges hegemonic norms, positioning neurodivergence as a political category alongside class, gender, and race (1).

Although these two approaches emerge from different contexts—applied disability research and social activism—both challenge medical views of autism and promote biopsychosocial, interactionist, and rights-based perspectives consistent with the principles of the UNCRPD. Yet, while models grounded in supports for QoL operationalize *what matters* and *how to get there* across systems, the neurodiversity paradigm underscores *for whom* and *on whose terms.* Despite shared aims, bringing the two perspectives into dialogue also reveals several conceptual tensions and blind spots.

The aim of this mini review is to adopt a critical stance examining how these perspectives intersect and diverge, and by considering how their tensions can be used productively to inform socio-political and paradigmatic debates, guide the design of supports and interventions, and future research. This is not intended as a systematic review but as a concise, integrative overview that draws on peer-reviewed articles and relevant scholarly sources from the past decade, including early foundational works where necessary.

## 2 Paradigmatic and socio-political perspectives

Language and terminology play a central role in shaping attitudes, values, and social practices. Within QoL-based approaches in the disability field, the model developed by Schalock and Verdugo (9) has become one of the most influential frameworks (10). This model is aligned with the current definition of intellectual and developmental disabilities (IDD), terminology that denotes a combined field encompassing intellectual disability (ID) and related developmental conditions (5). From this perspective, due to limitations in daily life, which may or may not include difficulties in intellectual functioning, autism is understood functionally within the broader field of developmental disabilities, with the aim of providing person-centered supports and guiding inclusive policies and services (11, 12). From this same perspective, disability is understood as an aspect of human experience rather than a deficit, and person-first language (*person with autism*) separates the condition from identity (11). In contrast, the neurodiversity paradigm promotes identity-first language (*autistic person*), viewing autism as an integral part of selfhood (13). Positioning language is the cornerstone for self-representation and political mobilization. From this standpoint, conceptualizing autism within the broader field of IDD could be seen as a deficit-oriented, with the potential to perpetuate ableist and stigmatizing assumptions about autistic individuals (14, 15).

Language tension extends to representation. The neurodiversity movement has been largely shaped by verbally communicative adults with strong self-reflection and advocacy abilities, many of whom received a late diagnosis and whose experiences challenge traditional diagnostic stereotypes (16). This self-advocacy movement is a source of collective pride and has been described as an autistic liberation (17). This raises the question of whether autistic people with extensive support needs, such as those with ID, non-speaking autistic individuals, or those with mental health conditions, are adequately represented within the neurodiversity paradigm and of who legitimately speaks for them. In principle, the paradigm encompasses people with extensive support needs and other neurominorities (18), although some authors argue that such inclusion is conceptually problematic (15). In practice, such voices remain typically scarce from research and public discourse. For instance, recent reviews of autistic identity indicate a striking underrepresentation of participants with ID, only 11 of 3,650 across published datasets (19), with other studies rarely disclosing this information (20). This underrepresentation is particularly relevant given that the impact of diagnosis and identity on well-being is among the most extensively studied topics in the neurodiversity literature (20, 21).

Accordingly, QoL represents a complex construct comprising multiple aspects of human experience aimed at achieving personal well-being, articulated through a series of dimensions such as social inclusion, emotional well-being, and interpersonal relationships, among others (12). Nevertheless, it is essential to consider what constitutes a good life from the perspective of autistic individuals, their families, and the standards by which they define it (22–24). On the other hand, from the neurodiversity perspective, the goal is precisely to contribute to this reorientation by foregrounding lived experience, self-advocacy, and identity pride (7). However, while this perspective offers significant advances, its relatively recent development has not yet articulated the conceptual, measurement, and applied structure that has characterized QoL frameworks in the field of IDD to promote inclusion and well-being (10).

## 3 Applied and clinical perspectives

The heterogeneity of autism poses particular challenges for clinical practice, requiring approaches that accommodate diverse needs and understandings of well-being. Both QoL models and the neurodiversity paradigm call for a rethinking of intervention methods, emphasizing processes that foster the recognition and empowerment of autistic people and their families rather than standardization. QoL models enable transformations at interconnected levels, from organizational planning to guiding professional evaluation and service design, ultimately shaping personalized supports that enhance outcomes in multiple life domains (5, 25). Similarly, neuroaffirmative approaches have emerged within the neurodiversity paradigm as a framework that reshapes applied practice by reinforcing respect for neurological differences and challenging normative assumptions that frame autism as deficit (26, 27). In clinical contexts, this translates into designing interventions that prioritize authenticity, recognition of individual capabilities, and a critical stance toward normalizing practices (28, 29).

A critical consideration in clinical practice is access to supports and services, which still depends largely on diagnostic processes. Yet obtaining a diagnosis continues to involve significant barriers for many (30). In this context, self-identification and self-diagnosis have frequently emerged as a response to exclusion from formal diagnostic pathways (31). Some neurodivergent voices therefore argue that formal diagnosis may be unnecessary, as autism can be assumed as a self-defined identity (32). Yet such positions raise concerns, including the “colonization” of the term, its use as an empty slogan, the romanticization of autism, as well as the neglect of the diverse support needs experienced by many autistic people. While recognizing strengths is valuable, autism is characterized by a wide range of cognitive profiles, communication styles, and support needs, dimensions often obscured by prevailing representations privileging autistic people with fewer adaptive-functioning challenges and depicting them as exceptionally gifted or as possessing “savant skills” (33). Many autistic people and their families face substantial challenges in daily functioning, and evidence consistently shows high rates of mental health problems and elevated suicide risk (34, 35). Such realities underscore the importance of effective and inclusive approaches that respond to the broad spectrum of needs, making diagnosis particularly relevant for accessing appropriate support.

Within this applied context, the provision of supports remains a cornerstone of QoL-based frameworks. Supports are defined as the resources and strategies—personal, social, and contextual—that promote development, education, interests, and well-being (25), enabling participation and autonomy, and translating rights into everyday practice. For autistic individuals with extensive support needs, they are central to achieving inclusion and safety, and enhance individual and family functioning (36). Yet supports can also be perceived as instruments of adaptation to a neurotypical environment rather than means for empowerment (15). Some neurodiverse perspectives recognize their value in fostering self-determination (1), but the key question is not whether supports are needed, but what kinds of supports are appropriate. From a neuroaffirmative approach, supports may stem from self-directed management that enhances self-care and personal agency, in combination with context that validate autistic needs as legitimate and facilitate sensory and emotional regulation (26). However, relying solely on self-managed strategies may also risk isolation or insufficient assistance. Risks persist when supports are misaligned: stigmatizing, insufficiently individualized, or grounded in assumptions that undervalue capacity due to differences in communication or functioning. The issue of how supports are conceptualized and delivered extends across the lifespan, including early interventions, where it is essential to examine the aims and risks of supports and how information is communicated to families (29, 37).

Finally, identity-focused interventions have become increasingly relevant for understanding well-being in autism. While neuroaffirmative approaches understand autism as a socially marginalized identity, they emphasize the importance of promoting a positive autistic identity as a key therapeutic goal linked to well-being and mental health (28). In practice, identity-affirming approaches aim to foster acceptance, belonging, and authenticity through therapeutic and educational interventions (38). Similarly, QoL frameworks have long incorporated emotional well-being, self-determination, empowerment, and positive self-concept, providing structured mechanisms to reinforce inclusion and participation (36). However, affirmative or autistic-pride interventions have been more visible among neurodivergent individuals without ID or with less pronounced autistic traits (20), which may lead families and support providers to regard identity-focused principles as less applicable—or more difficult to prioritize—in cases of individuals with extensive support needs and limited daily functioning. This highlights a tension between identity-based perspectives and support-oriented priorities, particularly in contexts where everyday care demands and functional challenges shape how well-being is understood and pursued.

## 4 Research perspectives

Despite decades of biomedical research, many autistic people remain critical of etiological approaches that prioritize identifying the underlying causes or treating (39). The limited explanatory power of such studies has done little to improve daily life outcomes, yet they continue to dominate funding and publication trends (8), reflecting the persistence of medical frameworks in which the primary goal has traditionally been to detect and eliminate the presumed genetic causes of disability (40). From a genetic standpoint, studies have focused on *de novo* heterozygous mutations, which show a strong association with autism (41). More than one hundred genes have been identified, yet these mutations account for only 10–20% of cases (41). Similarly, neuroimaging studies using MRI have yielded inconclusive results, although research on the so-called “social brain” may offer insight into the neurological bases of autism (42). In contrast, the autistic community generally values studies that have a tangible impact on their everyday life and inclusion. In this sense, recent analyses show that current research priorities often fail to reflect autistic people’s lived needs and experiences (43). Autistic adults, families, and those involved in providing supports consistently prioritize lifelong supports and well-being, yet these perspectives remain underrepresented in research agendas (43, 44). The literature highlights key areas, including improved access to healthcare and diagnosis, promotion of mental health and well-being, greater public understanding of autism, access to education and employment, and co-design research and supports with autistic collaborators (21, 39).

QoL models are particularly relevant for reorienting autism research toward these participatory priorities and inclusion (21, 22, 45). Within the QoL tradition, autism research has mainly focused on health-related and other objective components (46). Evidence consistently shows lower QoL outcomes among autistic people compared to the general population, often linked to factors such as poor mental health (47–49). However, these studies frequently rely on proxy informants, since the lived experiences of people with IDD are often mediated by families, institutions, or support providers (46, 49). This limits the extent to which findings represent the authentic perspectives of autistic individuals. In response, disability research increasingly adopts mixed-methods, inclusive and participatory approaches, incorporating co-design and the involvement of co-researchers with IDD (50). This aligns with the self-advocacy and identity-affirming orientation of the neurodiversity paradigm, although empirical evidence in this area remains limited. There has been a growing body of participatory research involving autistic people in the design of services, policies, and studies that affect them (51); however, the central challenge remains ensuring genuine participation of autistic people with extensive support needs (52). Continued research is needed to establish criteria for neuroaffirmative approaches, including in research contexts, and strengthen their empirical foundations (53).

Recognizing autistic individuals as knowledge holders is essential for advancing more ethical and impactful research (54). Individuals in marginalized positions often possess insights that are more accurate and relevant to their lived realities than those generated from privileged standpoints (55). Integrating these perspectives not only challenges deficit-based assumptions but also strengthens the scientific and social validity of autism research, ensuring meaningful contributions to inclusion and well-being.

## 5 Discussion

This mini review constitutes an initial step toward offering guidance for practitioners, support providers, researchers, and policymakers on how to bring together QoL and neurodiversity perspectives for autistic people across the spectrum of support needs. When considered alongside QoL perspectives, the neurodiversity approach contributes valuable insights regarding self-representation and identity, emphasizing internal processes of self-awareness and empowerment articulated from within the autistic experience. This helps enhance attention to subjective experiences and self-reported outcomes, allowing for a more accurate understanding of what constitutes a good life for autistic individuals with extensive support needs (1, 46). Conversely, when compared with QoL models, the neurodiversity paradigm still requires conceptual refinement to articulate a clear and operational definition of what constitutes neuroaffirmative practices. It should also broaden its representational frameworks to reflect the full heterogeneity of the spectrum, as previous experiences suggest this is both possible and necessary (56). The central challenge ahead lies in integrating identity-based understandings of autism with frameworks that promote equality without re-emphasizing difference.

In developing this synthesis and identifying the key tensions that emerge across the three perspectives examined, a set of principles is outlined that may support future work and contribute to ongoing dialogue in the field. Continued theoretical refinement and empirical research will be essential to further develop, validate, and operationalize these directions. Accordingly, these principles are proposed:

- Well-being depends not only on self-acceptance but also on the quality and responsiveness of available supports (20, 23). Identity and supports should be understood as mutually reinforcing well-being and inclusion.
- Without privileging a single position, the key issue regarding language lies in its contextual performativity (e.g., contexts related to access to supports), alongside respect for individual preferences (e.g., speaking in identity-based terms), while recognizing that the use of community-preferred terminology over diagnostic labels does not compromise scientific rigor (57).
- The value of identity lies not only in language but in its transformative potential. The key is not to contrast identity- and diagnosis-based understandings, but to develop diagnostic practices that are inclusive, participatory, and non-deficit oriented, recognizing both formal and self-identified pathways to autistic identity (31).
- The value of supports lies in their role as indispensable means for participation and inclusion (36, 45), not as a threat to identity. Recognizing support needs does not entail pathologization but an acknowledgement that, as in other human conditions, certain functional needs require individualized strategies (53).
- Supports and interventions should promote well-being and inclusion through flexible communication tools, valuing internal experiences, and acknowledging the impact of diagnosis on identity, while addressing environmental and attitudinal barriers that shape participation. They should reduce stigma, strengthen self-acceptance, and enhance belonging and inclusion (27). Early implementation is key, avoiding forced normalization and instead fostering growth toward optimal functioning (37).
- Inclusive and participatory research should ensure the genuine involvement of autistic people across the spectrum as co-producers of knowledge throughout all stages, guaranteeing accessibility and diverse forms of representation (52, 54). Future research and practice should prioritize issues that directly affect autistic preferences.

Finally, building truly inclusive communities requires embracing interdependence as a shared human condition. Differences enrich our collective experience but can also deepen the vulnerability of those in less privileged contexts. Rights become real not only through acceptance but through the tangible supports that enable participation and well-being for autistic individuals across the spectrum of support needs. In this sense, both perspectives invite us to move beyond diagnostic labels and to understand autism through the interaction between identity, needs, supports, and context, offering complementary frameworks to advance toward a more just society grounded in inclusion and social justice.

## Author positionality statement

The authors include neurodivergent scholars and practitioners working in the field of quality of life and disability supports. Our lived experience and professional backgrounds inform our interpretation of the literature and the integrative principles proposed in this review.

## References

[1] Grummt M . Sociocultural perspectives on neurodiversity—An analysis, interpretation and synthesis of the basic terms, discourses and theoretical positions. Sociol Compass. (2024) 18:e13249. doi: 10.1111/soc4.13249

[2] United Nations . Convention on the rights of persons with disabilities (2006). Available online at: https://www.ohchr.org/en/instruments-mechanisms/instruments/convention-rights-persons-disabilities (Accessed November 13, 2025).

[3] WHOQOL Group . The World Health Organization quality of life assessment (WHOQOL): Position paper from the World Health Organization. Soc Sci Med. (1995) 41:1403–9. doi: 10.1016/0277-9536(95)00112-K, PMID: 8560308

[4] Verdugo MA Navas P Gómez LE Schalock RL . The concept of quality of life and its role in enhancing human rights in the field of intellectual disability. J Intellect Disabil Res. (2012) 56:1036–45. doi: 10.1111/j.1365-2788.2012.01585.x, PMID: 22672317

[5] Schalock RL Luckasson R Tassé MJ . An overview of intellectual disability: definition, diagnosis, classification, and systems of supports (12th ed.). Am J Intellect Dev Disabil. (2021) 126:439–42. doi: 10.1352/1944-7558-126.6.439, PMID: 34700345

[6] Silverman S . Neurotribes: The legacy of autism and the future of neurodiversity. New York City: Penguin Random House (2015). 534 p.

[7] Chapman R Carel H . Neurodiversity, epistemic injustice, and the good human life. J Soc Philosophy. (2022) 53:614–31. doi: 10.1111/josp.12456

[8] Pellicano E den Houting J . Annual research review: Shifting from ‘normal science’ to neurodiversity in autism science. J Child Psychol Psychiatry. (2022) 63:381–96. doi: 10.1111/jcpp.13534, PMID: 34730840 PMC9298391

[9] Schalock RL Verdugo MA Braddock DL . Handbook on quality of life for human service practitioners 40. DC: American Association on Mental Retardation Washington (2002).

[10] Amor AM Verdugo MÁ Fernández M Aza A Sánchez-Gómez V Wolowiec Z . Development and validation of standardized quality of life measures for persons with IDD. Behav Sci (Basel). (2023) 13. doi: 10.3390/bs13060452, PMID: 37366704 PMC10295264

[11] Verdugo MA . Terminología y clasificación sobre discapacidades intelectuales y del desarrollo [Internet]. Salamanca: Universidad de Salamanca: Instituto Universitario de Integración en la Comunidad (INICO (2020). Available online at: https://sid-inico.usal.es/documentacion/terminologia-y-clasificacion-sobre-discapacidades-intelectuales-y-del-desarrollo/ (Accessed November 17, 2025).

[12] Schalock RL Verdugo Alonso MÁ . El concepto de calidad de vida en los servicios y apoyos para personas con discapacidad intelectual. Siglo Cero: Rev Española sobre Discapacidad Intelect. (2007) 38:21–36.

[13] Kenny L Hattersley C Molins B Buckley C Povey C Pellicano E . Which terms should be used to describe autism? Perspectives from the UK autism community. Autism. (2016) 20:442–62. doi: 10.1177/1362361315588200, PMID: 26134030

[14] Bottema-Beutel K Kapp SK Lester JN Sasson NJ Hand BN . Avoiding ableist language: Suggestions for autism researchers. Autism Adulthood. (2021) 3:18–29. doi: 10.1089/aut.2020.0014, PMID: 36601265 PMC8992888

[15] Jaarsma P Welin S . Autism as a natural human variation: Reflections on the claims of the neurodiversity movement. Health Care Anal. (2012) 20:20–30. doi: 10.1007/s10728-011-0169-9, PMID: 21311979

[16] Bargiela S Steward R Mandy W . The experiences of late-diagnosed women with autism spectrum conditions: An investigation of the female autism phenotype. J Autism Dev Disord. (2016) 46:3281–94. doi: 10.1007/s10803-016-2872-8, PMID: 27457364 PMC5040731

[17] Ryan S Milton D . The Routledge International Handbook of Critical Autism Studies. Routledge. New York: Taylor & Francis Group (2023).

[18] Grinker RR . Autism, “Stigma,” Disability: A shifting historical terrain. Curr Anthropol. (2020) 61:S55–67. doi: 10.1086/705748

[19] Huang Y Trollor JN Foley KR Arnold SRC . I’ve spent my whole life striving to be normal”: Internalized stigma and perceived impact of diagnosis in autistic adults. Autism Adulthood. (2023) 5:423–36. doi: 10.1089/aut.2022.0066, PMID: 38116050 PMC10726184

[20] Najeeb P Quadt L . Autistic well-being: A scoping review of scientific studies from a neurodiversity-affirmative perspective. Neurodiversity. (2024) 2:1–26. doi: 10.1177/27546330241233088

[21] McConachie H Wilson C Mason D Garland D Parr JR Rattazzi A . What is important in measuring quality of life? Reflections by autistic adults in four countries. Autism Adulthood. (2020) 2:4–12. doi: 10.1089/aut.2019.0008, PMID: 36600984 PMC8992842

[22] Merrington H Gibbs V Haas K Clark T Robinson A AlAnsari M . What matters most? An exploration of quality of life through the everyday experiences of autistic young people and adults. Autism Adulthood. (2025) 7:312–23. doi: 10.1089/aut.2023.0127, PMID: 40539216 PMC12174840

[23] Milton D Sims T . How is a sense of well-being and belonging constructed in the accounts of autistic adults? Disabil Soc. (2016) 31:520–34. doi: 10.1080/09687599.2016.1186529

[24] Battanta NK Jenni OG Schaefer C von Rhein M . Autism spectrum: parents’ perspectives reflecting the different needs of different families. BMC Pediatr. (2024) 24:439. doi: 10.1186/s12887-024-04912-x, PMID: 38982431 PMC11232266

[25] Thompson JR Bradley VJ Buntinx WHE Schalock RL Shogren KA Snell ME . Conceptualizing supports and the support needs of people with intellectual disability. Intellect Dev Disabil. (2009) 47:135–46. doi: 10.1352/1934-9556-47.2.135, PMID: 19368481

[26] Chapman R Botha M . Neurodivergence-informed therapy. Dev Med Child Neurol. (2023) 65:310–7. doi: 10.1111/dmcn.15384, PMID: 36082483

[27] Curnow E Utley I Rutherford M Johnston L Maciver D . Diagnostic assessment of autism in adults – Current considerations in neurodevelopmentally informed professional learning with reference to ADOS-2. Front Psychiatry. (2023) 14:1258204. doi: 10.3389/fpsyt.2023.1258204, PMID: 37867776 PMC10585137

[28] Pantazakos T Vanaken GJ . Addressing the autism mental health crisis: The potential of phenomenology in neurodiversity-affirming clinical practices. Front Psychol. (2023) 14:1225152. doi: 10.3389/fpsyg.2023.1225152, PMID: 37731874 PMC10507173

[29] Leadbitter K Buckle KL Ellis C Dekker M . Autistic self-advocacy and the neurodiversity movement: Implications for autism early intervention research and practice. Front Psychol. (2021) 12:635690. doi: 10.3389/fpsyg.2021.635690, PMID: 33912110 PMC8075160

[30] Scattoni ML Micai M Ciaramella A Salvitti T Fulceri F Fatta LM . Real-world experiences in autistic adult diagnostic services and post-diagnostic support and alignment with services guidelines: Results from the ASDEU study. J Autism Dev Disord. (2021) 51:4129–46. doi: 10.1007/s10803-021-04873-5, PMID: 33502713 PMC8510906

[31] Friedman A Paltoglou A Sorte R . A qualitative exploration of the experiences of self-diagnosed autistic women and gender-diverse individuals who are not pursuing an autism diagnosis. Neurodiversity. (2024) 2:1–13. doi: 10.1177/27546330241307828

[32] Lewis LF . Exploring the experience of self-diagnosis of autism spectrum disorder in adults. Arch Psychiatr Nurs. (2016) 30:575–80. doi: 10.1016/j.apnu.2016.03.009, PMID: 27654240

[33] Brown MI Fisher HR . Promoting neurodiversity without perpetuating stereotypes or overlooking the complexity of neurodevelopmental disorders. Ind Organizational Psychol. (2023) 16:36–40. doi: 10.1017/iop.2022.97

[34] Hirvikoski T Mittendorfer-Rutz E Boman M Larsson H Lichtenstein P Bölte S . Premature mortality in autism spectrum disorder. Br J Psychiatry. (2016) 208:232–8. doi: 10.1192/bjp.bp.114.160192, PMID: 26541693

[35] Lai MC Kassee C Besney R Bonato S Hull L Mandy W . Prevalence of co-occurring mental health diagnoses in the autism population: A systematic review and meta-analysis. Lancet Psychiatry. (2019) 6:819–29. doi: 10.1016/S2215-0366(19)30289-5, PMID: 31447415

[36] Gómez LE Schalock RL Verdugo MÁ . A quality of life supports model: Six research-focused steps to evaluate the model and enhance research practices in the field of IDD. Res Dev Disabil. (2021) 119. doi: 10.1016/j.ridd.2021.104112, PMID: 34655955

[37] Manzini A Jones EJH Charman T Elsabbagh M Johnson MH Singh I . Ethical dimensions of translational developmental neuroscience research in autism. J Child Psychol Psychiatry. (2021) 62:1363–73. doi: 10.1111/jcpp.13494, PMID: 34405894 PMC7611913

[38] Crane L Hearst C Ashworth M Davies J Hill EL . Supporting newly identified or diagnosed autistic adults: An initial evaluation of an autistic-led programme. J Autism Dev Disord. (2021) 51:892–905. doi: 10.1007/s10803-020-04486-4, PMID: 32266684 PMC7954714

[39] Cage E Botha M McDevitt L King KN Biscoe L Tucker K . Diagnosis as a new beginning not an end: A participatory photovoice study on navigating an autism diagnosis in adulthood. Autism. (2024) 28:2014–27. doi: 10.1177/13623613231220418, PMID: 38230649

[40] Brock DW . Preventing genetically transmitted disabilities while respecting persons with disabilities. In: Wasserman D Bickenbach J Wachbroit R , editors. Quality of Life and Human Difference: Genetic Testing, Health Care, and Disability. Cambridge University Press, Cambridge (2005). p. 67–100. (Cambridge Studies in Philosophy and Public Policy).

[41] Iossifov I O’Roak BJ Sanders SJ Ronemus M Krumm N Levy D . The contribution of *de novo* coding mutations to autism spectrum disorder. Nature. (2014) 515:216–21. doi: 10.1038/nature13908, PMID: 25363768 PMC4313871

[42] Elsabbagh M Johnson MH . Autism and the social brain: The first-year puzzle. Biol Psychiatry. (2016) 80:94–9. doi: 10.1016/j.biopsych.2016.02.019, PMID: 27113503

[43] Roche L Adams D Clark M . Research priorities of the autism community: A systematic review of key stakeholder perspectives. Autism. (2021) 25:336–48. doi: 10.1177/1362361320967790, PMID: 33143455

[44] Clark M Adams D . Listening to parents to understand their priorities for autism research. PloS One. (2020) 15:e0237376. doi: 10.1371/journal.pone.0237376, PMID: 32790720 PMC7425861

[45] Tobin MC Drager KDR Richardson LF . A systematic review of social participation for adults with autism spectrum disorders: Support, social functioning, and quality of life. Res Autism Spectr Disord. (2014) 8:214–29. doi: 10.1016/j.rasd.2013.12.002

[46] Evers K Maljaars J Schepens H Vanaken GJ Noens I . Conceptualization of quality of life in autistic individuals. Dev Med Child Neurol. (2022) 64:950–6. doi: 10.1111/dmcn.15205, PMID: 35323990

[47] Ayres M Parr JR Rodgers J Mason D Avery L Flynn D . A systematic review of quality of life of adults on the autism spectrum. Autism. (2018) 22:774–83. doi: 10.1177/1362361317714988, PMID: 28805071

[48] Oakley BF Tillmann J Ahmad J Crawley D San José Cáceres A Holt R . How do core autism traits and associated symptoms relate to quality of life? Findings from the longitudinal european autism project. Autism. (2021) 25:389–404. doi: 10.1177/1362361320959959, PMID: 33023296 PMC7874383

[49] Van Heijst BF Geurts HM . Quality of life in autism across the lifespan: A meta-analysis. Autism. (2015) 19:158–67. doi: 10.1177/1362361313517053, PMID: 24443331

[50] Shogren KA . The right to science: Centering people with intellectual disability in the process and outcomes of science. Intellect Dev Disabil. (2023) 61:172–7. doi: 10.1352/1934-9556-61.2.172, PMID: 36996278

[51] Fletcher-Watson S Adams J Brook K Charman T Crane L Cusack J . Making the future together: Shaping autism research through meaningful participation. Autism. (2019) 23:943–53. doi: 10.1177/1362361318786721, PMID: 30095277 PMC6512245

[52] de Haas C Grace J Hope J Nind M . Doing research inclusively: Understanding what it means to do research with and alongside people with profound intellectual disabilities. Soc Sci. (2022) 11. doi: 10.3390/socsci11040159

[53] Dwyer P . The neurodiversity approach(es): What are they and what do they mean for researchers? Hum Dev. (2022) 66:73–92. doi: 10.1159/000523723, PMID: 36158596 PMC9261839

[54] Thurm A Halladay A Mandell D Maye M Ethridge S Farmer C . Making research possible: Barriers and solutions for those with ASD and ID. J Autism Dev Disord. (2022) 52:4646–50. doi: 10.1007/s10803-021-05320-1, PMID: 34716842 PMC9054938

[55] Gillespie-Lynch K Kapp SK Brooks PJ Pickens J Schwartzman B . Whose expertise is it? Evidence for autistic adults as critical autism experts. Front Psychol. (2017) 8:438. doi: 10.3389/fpsyg.2017.00438, PMID: 28400742 PMC5368186

[56] Baggs M . Losing. In: Kapp SK , editor. Autistic Community and the Neurodiversity Movement: Stories from the Frontline. Singapore: Springer Nature (2020). p. 77–86.

[57] Bottema-Beutel K Yoder PJ Hochman JM Watson LR . The role of supported joint engagement and parent utterances in language and social communication development in children with Autism Spectrum Disorder. J Autism Dev Disord. (2014) 44:2162–74. doi: 10.1007/s10803-014-2092-z, PMID: 24658867 PMC4171249

